# Ten simple rules for quick and dirty scientific programming

**DOI:** 10.1371/journal.pcbi.1008549

**Published:** 2021-03-11

**Authors:** Gabriel Balaban, Ivar Grytten, Knut Dagestad Rand, Lonneke Scheffer, Geir Kjetil Sandve

**Affiliations:** 1 Biomedical Informatics Group, Department of Informatics, University of Oslo, Oslo, Norway; 2 PharmaTox Strategic Research Initiative, Faculty of Mathematics and Natural Sciences, University of Oslo, Oslo, Norway; 3 Institute of Medical Microbiology, Oslo University Hospital, Rikshospitalet, Oslo, Norway; Dassault Systemes BIOVIA, UNITED STATES

This is a *PLOS Computational Biology* Software paper.

## Introduction

Any textbook or course in programming will tell you to write programs that are well structured, well documented, and thoroughly tested, to ensure correctness and ease of maintenance. However, when faced with time pressures and the eagerness of reaching a scientific result, you may instead find yourself coding in a quick and dirty style that does not live up to the ideal. Indeed, coding quickly can be a good way to deal with the uncertain and explorative nature of computational research. The quicker you code, the more scientific ideas you can potentially test and publish. However, if coding quickly means coding sloppily, then bugs, false conclusions, and article retractions [1] may be the result. Furthermore, if your code becomes increasingly complex and messy over time, then adapting it to new tasks will be difficult, potentially stalling your research progress. Effective scientific programming therefore requires a constant balance between the needs of the present and the needs of the future, with a coding style that is quick and dirty but rigorous enough.

In our guide, we focus on software development speed and formulate a set of 10 simple rules for writing good enough quality code with minimal effort, with the aim to increase research productivity. We follow in the tradition of promoting software competence among scientists [2–4] and build on previous installments in the Ten Simple Rules Series, which aim to make scientific software more robust [5], useable [6], reproducible [7], open [8], and effective [9].

Our rules are based on our personal experiences with scientific software development, and on industrial software methodologies [10–14], which we have applied to various computational biology research projects over the last decade. While we found that many software industry practices were applicable to our research, we also experienced that they could not be applied blindly and needed some adaptation. This is because scientific software development is typically done by relatively few individuals, who are also domain experts and intimately familiar with the software requirements. In contrast, the industrial setting most often involves large teams of developers who rely on separate business experts to define their goals. We therefore focus on the subset of industrial software engineering practices that we found most useful, and bring them to you in a condensed form.

**Fig 1:**
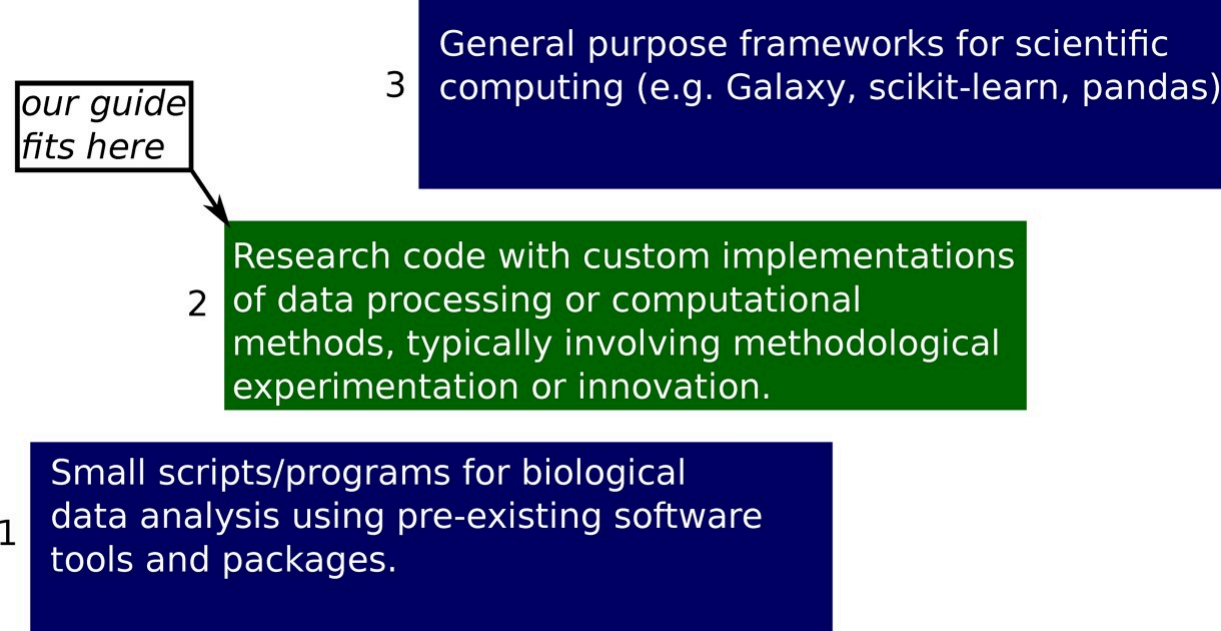
Levels of scientific software engineering sophistication in computational biology.

Software engineering requirements can vary greatly in computational biology applications (Fig 1), ranging from simple scripts for routine data analysis with preexisting tools and software packages, up to sophisticated general purpose frameworks [15–17], which seek to automate and simplify a wide range of computing tasks. We kindly refer readers learning to program to [18] and readers working on large general purpose scientific computing frameworks to the software engineering literature [10–14].

Our advice is intended for researchers who already know how to code and are working on focused scientific projects that require experimentation with computational and or data processing methods. Throughout our guide, we assume familiarity with basic Software Carpentry concepts [4], such as version control [19] and data management [20].

### Rule 1: Think before you code

“*Preparation*, *I have often said*, *is rightly two-thirds of any venture*.*”—Amelia Earhart*

Computer programming is a world of details and decisions, and it is good to take stock of where you are before you enter. Why are you coding and how does your code help you to achieve your scientific goals? Once an idea pops up, it can be tempting to just start working, since the process of programming provides a feeling of producing something. However, constantly doing something does not necessarily mean that you are doing the right things.

Try to get as much relevant information as you can before starting to write code, as suggested in [9]. This should include a literature review of the computational methods and application domain that you will be working with, as well as a search for preexisting software implementations which you can use in your project. Also, consider the expertise of other people in your team or community and whether there are any potential collaborations which would benefit your project. Organize your relevant information into sketches, mind maps, wikis, or lists which you can consult regularly to see the big picture. A few hours of literature review or discussion with colleagues can save you weeks of programming and should not be underestimated. Also, a good knowledge of your research topic and the people that work in it will allow you to make better technical decisions, leading to simpler software designs and effort invested in the right places.

If you are working with datasets, try to become aware of the strengths and weaknesses of your data as early as possible. This is especially relevant if your data was collected by collaborators, who may have important knowledge that you are currently unaware of. We recommend that you start your data analysis with simple exploratory techniques, such as summary statistics, histograms, and pair-plots, to quickly get an overview. Such information is useful for discussions with collaborators and for informing further analysis.

### Rule 2: Start with prototypes and expand them in short development cycles

“*Do the simplest thing that could possibly work*.*”—Kent Beck*

Research projects are like start-up businesses in that they often rely on a critical set of assumptions. For example, a typical better methods research project assumes that the existing methods can be improved upon, whereas an applied project assumes that publishable interpretations can be made from the analysis of some data. It is important to test your key assumptions as early as possible before committing too much of your time and effort. In the computational setting, this can be accomplished by prototyping techniques.

The start-up literature [21] recommends the use of a Minimum Viable Product (MVP), the smallest program (in terms of required effort) you need to test your key project assumptions. Such a prototype can provide you with valuable information while minimizing the time invested in it, thereby allowing you to decide whether a research project is worth pursuing as it is or whether it needs to be modified or dropped. If a project is worth pursuing, then the prototype serves as a basis for future development and can provide valuable insights into the data, methods, and technologies that you are working with.

**Fig 2:**
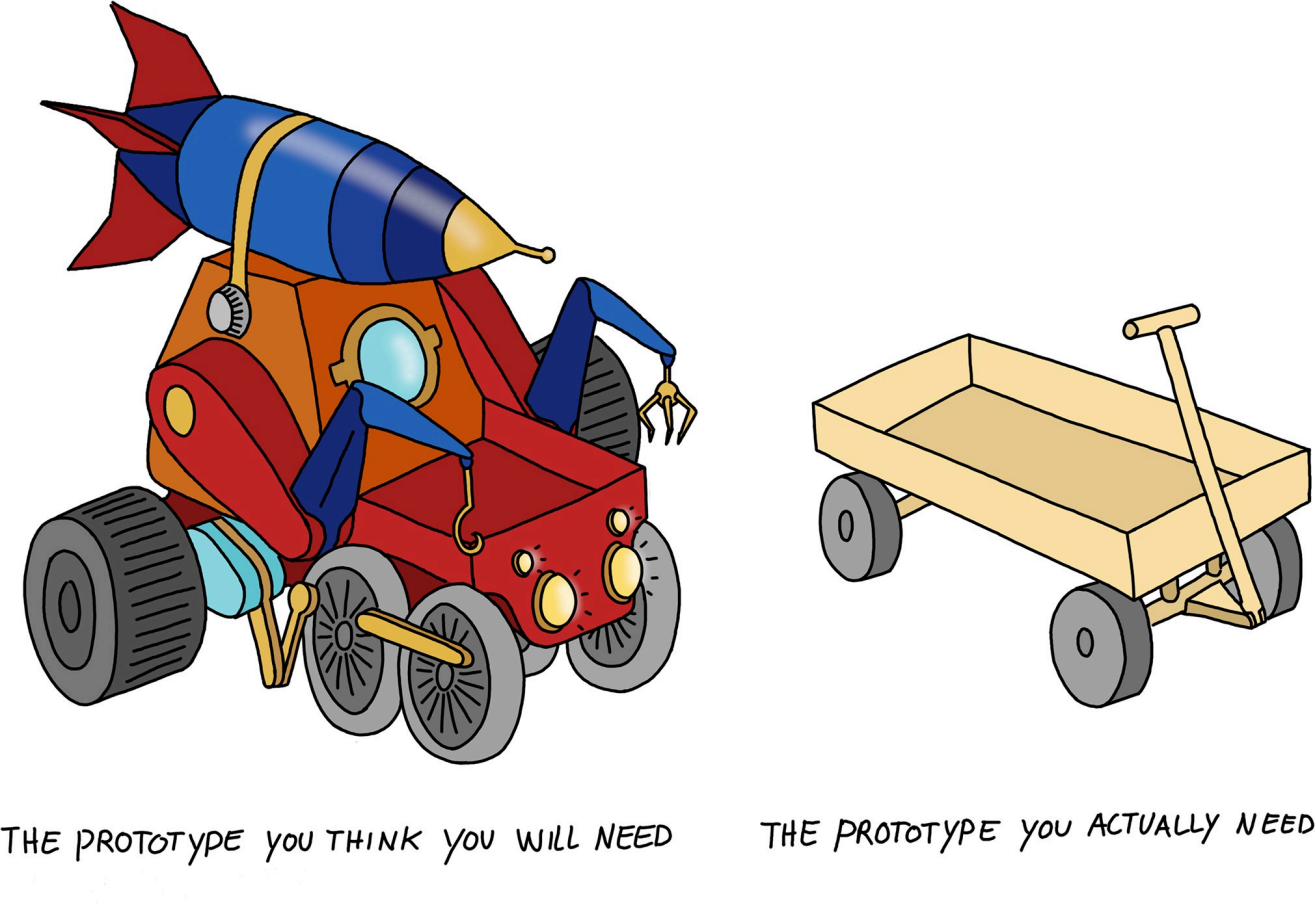
Minimize your prototypes by focusing on the core questions that you are trying to answer at the moment. Avoid implementing features speculatively for the future.

It can be tempting to implement a few extra features speculatively for the future at the prototype stage of your project. However, you should avoid doing this (Fig 2), as the best way to prepare for the future is to make the currently required code as clear and straightforward as possible [13,22]. To keep your prototype simple, reuse existing tools and libraries (see Rule 3) and create mocks [23] which simulate the outputs of code that you do not need to write at the moment. Mock code can always be replaced with full implementations later on. Also, using high-level programming languages (e.g., Python or R) can be very beneficial at this stage, as you can build your prototype more quickly and with less code than with low-level languages.

Once your prototype has been finished, you should assess what you have learned and use this information to guide what to do next. From here, we suggest that continued development should be done in short cycles, focusing on the current most critical assumptions that need to be tested. Each cycle should start with a plan to write the minimal set of code to reach the next goal and should end with a critical assessment of the results and useful information for what to do next. Such short development cycles allow you to frequently reassess the situation and keep you on the right track [21].

### Rule 3: Look for opportunities for code reuse

“*Measuring programming progress by lines of code is like measuring aircraft building progress by weight*.*”—Bill Gates*

**Fig 3:**
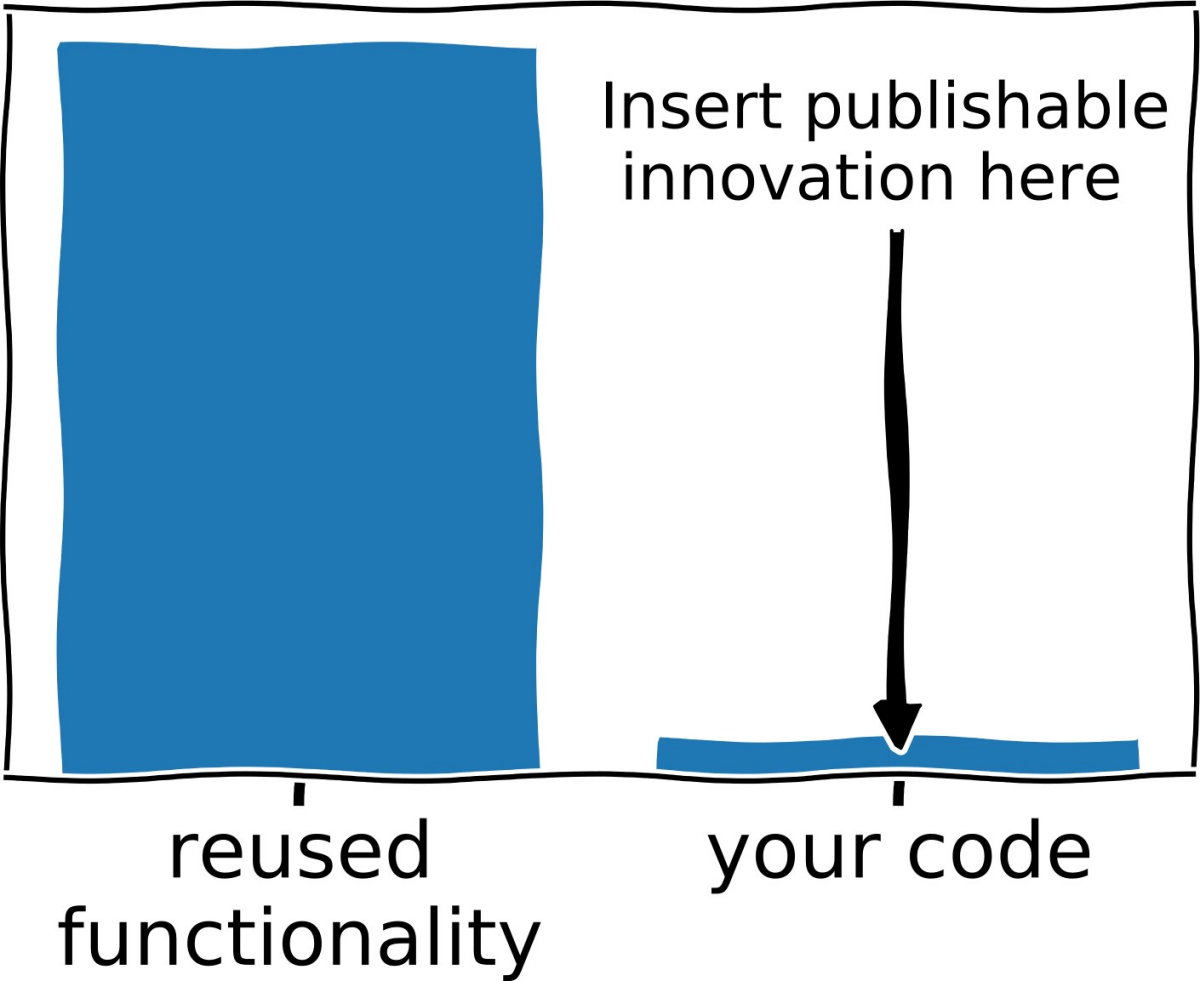
An ideal computational research project has a high amount of code reuse and a publishable innovation implemented in a small amount of new code.

Reuse of existing code can be a powerful way to keep development time short and get working solutions quickly (Fig 3). Code reuse comes in many forms, ranging from importing and using existing libraries, running existing tools, reusing your own code (see Rule 9), and cloning/extending existing code bases to simply copying short snippets of code that others have written. All of these ways of reusing existing code can potentially save you hours of development time. Beyond this immediate benefit, code reuse also centralizes or outsources maintenance of the reused code, potentially saving you time and effort in the future. For example, if you import and use the functionality from a well-known package such as scikit-learn [16] instead of developing your own code, you can be assured that this code will most likely gradually improve and be thoroughly maintained over the coming years.

**Fig 4:**
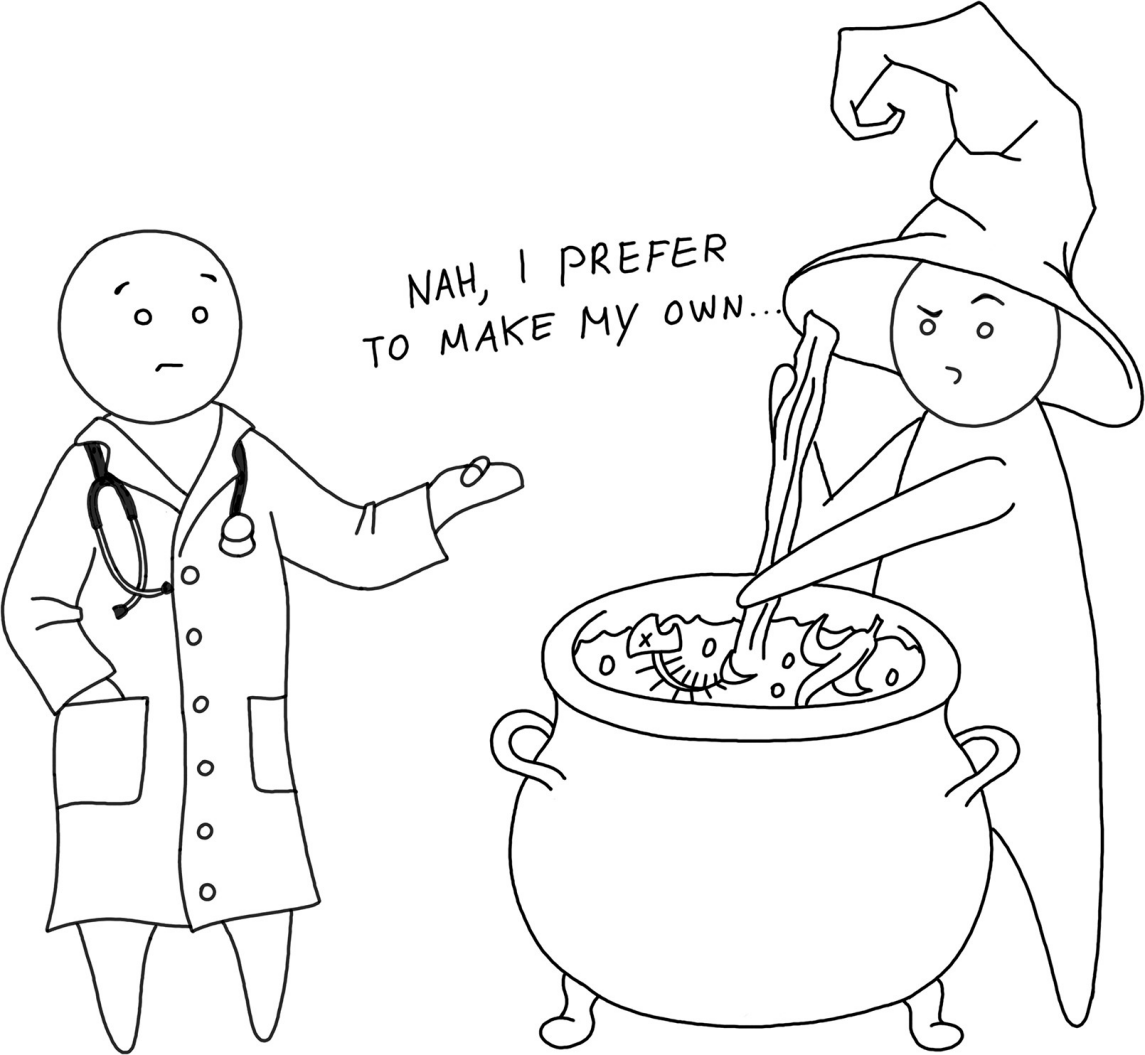
Not invented here syndrome.

It is human nature to trust your own code more than code written by somebody else. When this is taken to the extreme, it can result in the “not invented here” syndrome [24], an irrational preference for self-made solutions (Fig 4). Though you may be more comfortable working with something you wrote yourself, you should consider that well-established and regularly updated code bases are typically the result of countless hours of solving minor problems and edge-cases that you may not yet even be aware of, as well as a shared effort to remove nontrivial bugs. Reusing high-quality code bases can therefore save you from hitting the mistakes and pitfalls that have been previously solved by others.

On the other hand, implementing an algorithm yourself can be a fantastic way to broaden your knowledge, especially when you can compare your implementation to a preexisting reference. Also, writing your own code can allow you to avoid dependency issues associated with external packages (also known as dependency hell). You should therefore weigh the benefits of external packages against the expected effort of managing any dependencies. Thankfully, modern dependency management tools such as Packrat for R or pipenv for Python have made the job of managing dependencies much easier than in the past, allowing you to more comfortably make use of external packages in your code.

You can find opportunities for code reuse by browsing the literature, searching online, and talking to colleagues or other experts. When evaluating an external library or tool, consider the effort required to install it and integrate it with your own code, as well as whether any underlying assumptions hold in your case. A good indicator of library/tool quality is a substantial user base and recent development and/or updates. However, even if a tool or library is not a perfect fit, it may be worth using it in the early phases of your project to make progress. You can always replace the imperfect tool or library later on with something better.

### Rule 4: Modularize your code

“*The art of programming is the art of organizing complexity*, *of mastering multitude and avoiding its bastard chaos as effectively as possible*.*”—Edsger Dijkstra*

**Fig 5:**
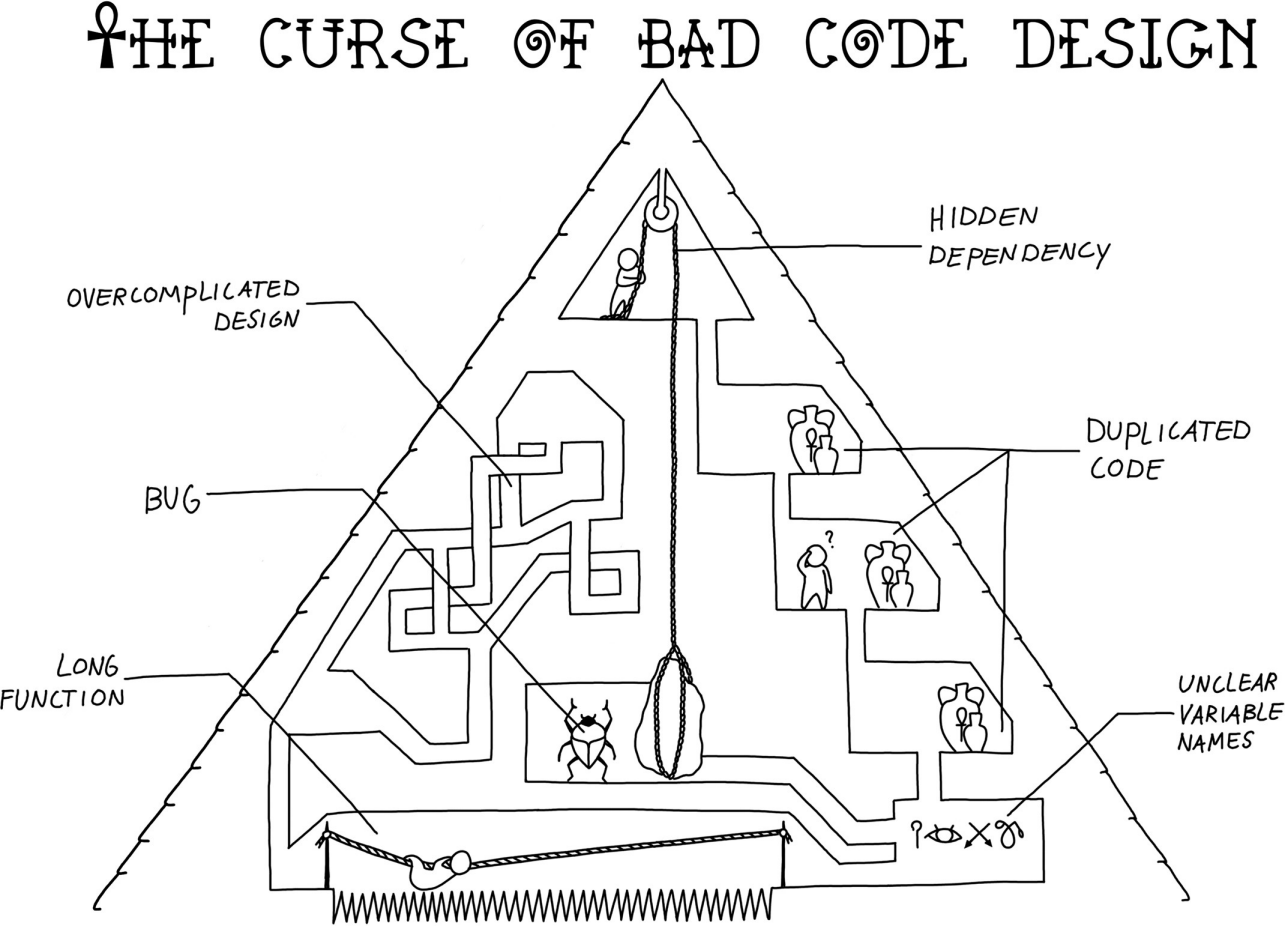
Working with a badly designed code base is difficult. Modularize your code to avoid getting lost and to prevent bugs.

Modularization is the process of organizing your code into reusable and parametrizable components. Even when you are trying to code quickly, modularization is a good idea; it prevents errors, makes your code more readable and testable, and facilitates code reuse over unnecessary duplication (Fig 5). With a bit of practice and a few guidelines, modularization can become second nature to you and will not slow down your coding.

A good way to begin with modularization is to observe the DRY (Do not Repeat Yourself) principle [10], which says to avoid unnecessary duplication of code. Reusable functions can be used instead of duplicate code, which also makes your code more readable and generalizable ([12]; Chapter 7). A simple rule of thumb is to not let your functions become more than a screen long. This will keep your functions manageable and focused on a specific task. A little bit of thinking is sometimes required when breaking up large functions. However, if you make it a regular habit, you will ultimately achieve simpler code designs with minimal effort.

If you are using an object-oriented language such as Python or C++, then you can further modularize your code with classes. An easy way to do this is to start with procedural code, and then group together related variables and functions into classes. Using object-orientation this way allows you to encapsulate technical details, better manage complexity, and more easily reuse code across projects. Beyond simply organizing functions and variables, object-oriented code can be made more sophisticated by exploiting polymorphic behavior, inheritance, object composition, and design patterns [25,26]. These strategies facilitate flexible code with highly dynamic run-time behavior.

That being said, there is always the risk of overengineering object-oriented programs, which results in overly complex code which is difficult to understand and modify. To avoid overengineering, you should always try first to avoid complexity before using advanced object-oriented strategies to deal with complexity. Be concrete with what you want to achieve and keep your scientific goals in mind. Finally, consider the advantages of programs based on low-level loops and arrays, which can be simpler and faster than object-oriented code.

The way that you modularize your code into functions and classes forms a high-level design of your code. We recommend that you let your design evolve as you code by adding functions and classes as you need them, rather than trying to develop your design a priori. When done properly, such an ad-hoc design process is very efficient and will give you only the modularization that you actually need ([13]; Chapter 17), which helps to prevent overengineering. Furthermore, “perfect” software designs can be very time consuming to develop and often require that existing functionality be rewritten many times. For most scientific applications, and especially for prototyping, a “good enough” design is all that is needed ([10]; Chapter 1).

### Rule 5: Avoid premature optimization

“*Premature optimization is the root of all evil*.*”—Donald Knuth*

**Fig 6:**
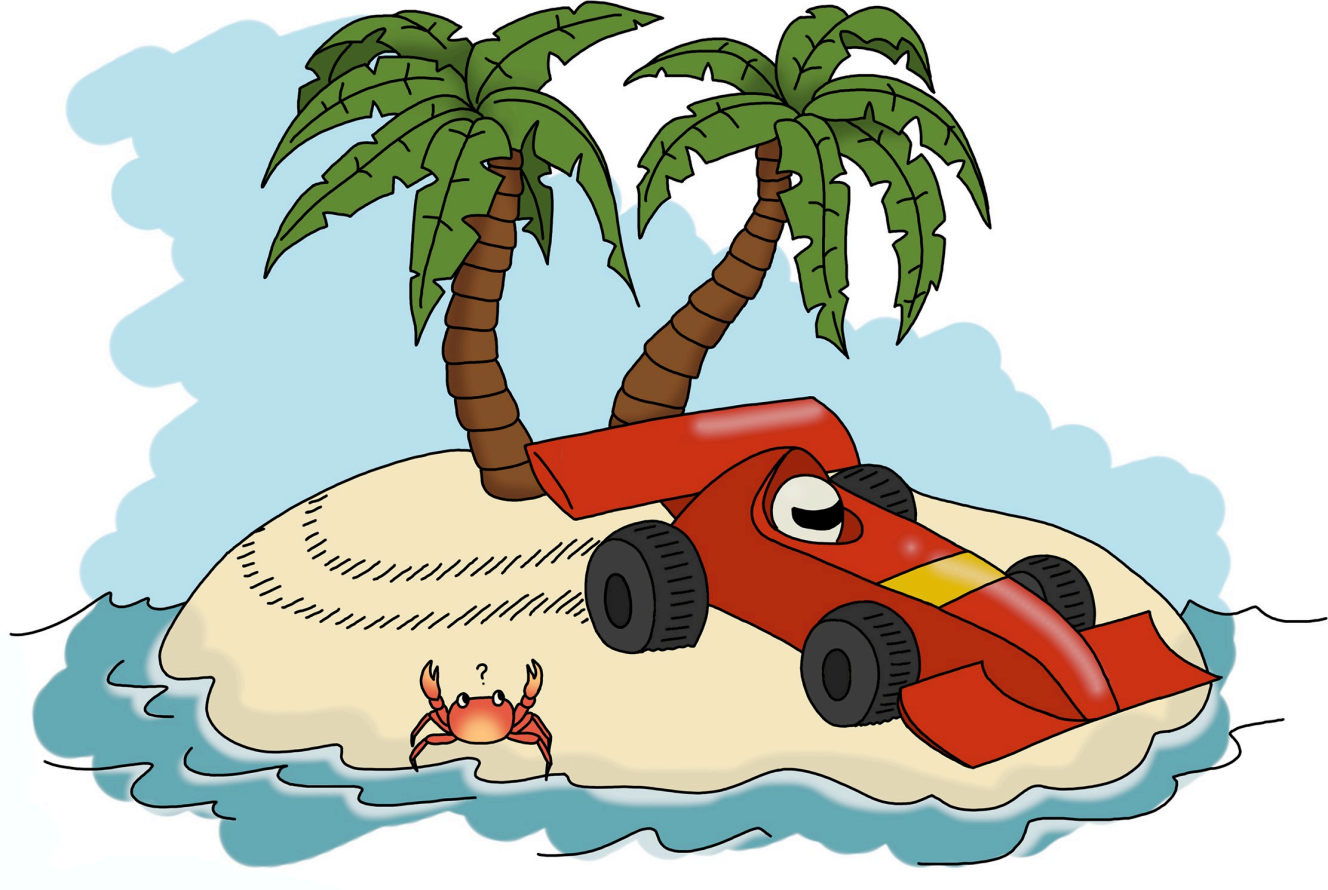
Premature optimization, avoid it like the plague.

It has become increasingly common to work with large datasets, which can greatly slow down the execution of computer programs. In these situations and any others where code is running slowly, it may be tempting to optimize the code to make it more efficient. However, optimizing code comes with risks: it can be time consuming, you might break the code, and you may end up making the code more complex or specialized in order to speed it up (Fig 6). Before you optimize code, you should consider the alternatives.

The best alternative to code optimization is reducing the size of the computation/data. If you are working with large datasets, try to subsample them or replace them with smaller datasets. In the early phases of a research project, small synthetic datasets are often the best way to go. Only scale up the input data when you are sure that your code does what you want. You may then find that you only need to analyze your large dataset a limited number of times, so that you may be able to live with the long run-time after all.

Another good alternative to code optimization is doing other things while a program with a long execution time runs. If the program runs for hours, then you can always catch up on the latest research articles while you wait. If the program takes a day or two, consider running it over the weekend or on a high-performance cluster. Your time is more valuable than your computer’s, and you should have a very good reason to trade your time spent on run-time optimization for your computer’s execution time.

If you end up pursuing run-time optimization, it is worthwhile to start by profiling your program to find the critical spots which are running slowly. While profiling, make sure your program inputs are sufficiently large, as some algorithms only become inefficient with a large enough problem size. Look for low-hanging algorithmic optimizations first, that is a better algorithm that reduces the number of operations that need to be performed. Before carrying out an optimization, have a test in mind that you can use to check that you have not introduced a bug. If your optimization is complex, use an automated unit test (see Rule 6). If you are using a high-level language such as Python or R and no better algorithm exists for your bottleneck, consider using a low-level language such as C. Look for existing wrappers to low-level code first, and only create your own low-level implementations as a last resort.

### Rule 6: Use automated unit testing for critical components

“*More than the act of testing*, *the act of designing tests is one of the best bug preventers known*.*”—Boris Beizer*

Unit tests are small pieces of code which test that a given function produces an expected output given a set of predetermined inputs. Unit tests are considered software engineering best practice [27], because they provide a quick and automated way for future developers (including yourself) to verify that your code has not been broken after an update.

Unit tests can play a valuable role in your exploratory and experimental scientific coding. This is because your code may change as a result of your varying research priorities, which may cause you to inadvertently introduce bugs. You would not want to abandon a good research direction because your “quick and dirty” code erroneously reported little to no signal. Conversely, reporting flawed results due to programming errors is bad science and has led to article retractions in the past [1]. Unit tests can help to avoid such problems, because they instantly make you aware of broken functionality before it becomes a problem.

**Fig 7:**
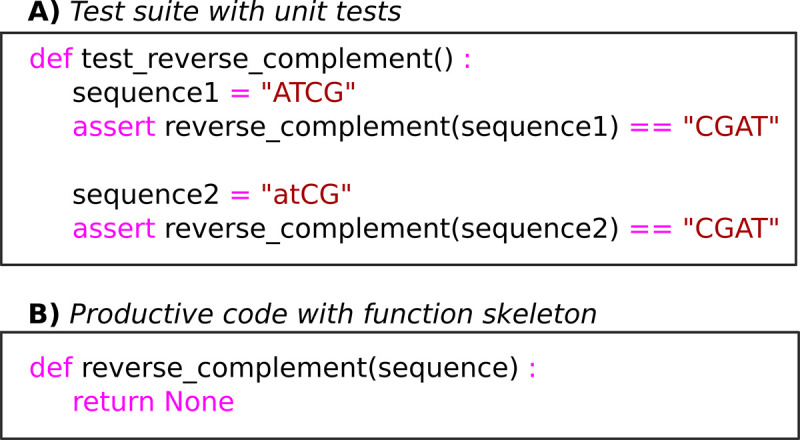
Demonstration of the test-first approach to writing automated unit tests. The desired function should calculate the reverse complement of a DNA sequence and also be case insensitive. (A) The test code in the top panel is written first, along with a skeleton reverse complement function in the bottom panel, which initially fails the test. (B) The reverse complement code can then be written and should pass its unit test when it is complete.

An easy way to get started with unit testing is to practice the “test-first approach,” which consists of designing a test before implementing productive code. In practice, before writing a function, you can write a small code piece that calls the function with some test data. This code piece can then be turned into an automated test by adding an assert statement to check if the function output meets some expected criteria. An example unit test demonstrating this idea is given in Fig 7. In order for your unit tests to be useful, they should be simple, quick to run, and give unambiguous error messages when they fail. Stochastic test data should be fixed with consistent seed values, to avoid randomness in unit test results.

**Fig 8:**
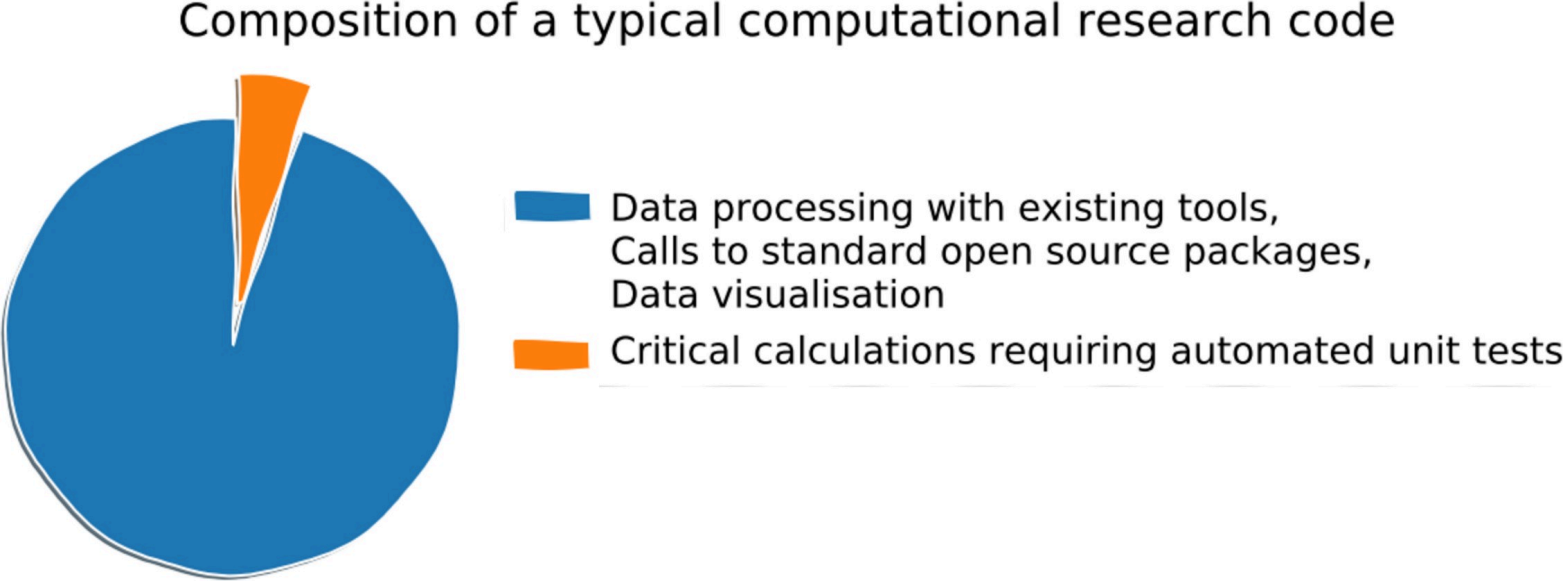
Many computational research projects contain just a few critical components responsible for important scientific calculations. Try to identify these critical components and write automated unit tests for them to avoid potential bugs and errors.

Writing and maintaining test code requires some effort, which is why we recommend you focus your testing on critical parts of your code which might produce errors with scientific consequences (Fig 8), rather than testing everything as per industry best practice. For example, miscalculating a *P* value is a potential problem, whereas an error in a progress bar is relatively harmless. Numerical optimization methods and partial differential equation solvers are good examples of code which should be tested automatically. Fortunately, the availability of good open-source scientific packages means that many critical calculations can be outsourced away from your project code, leaving only a small number of functions which may need to be verified by unit testing.

Note that if you work on a larger project with many components, you may also find it useful to automatically test several components together to see how they interact. Such tests are called integration tests. Depending on your project and your personality, it may be more convenient to write higher-level integration tests rather than lower-level unit tests. For maximum safety, use both.

### Rule 7: Refactor frequently

“*An ounce of prevention is worth a pound of cure*.*”—Benjamin Franklin*

Modern software development methodologies have embraced frequent changes in goals and requirements, and recommend the iterative evolution of a software system rather than the implementation of a prespecified and inflexible plan [14]. A consequence of this is the need for frequent refactoring, which is the process of changing a software system in such a way that it does not alter the external behavior of the code yet improves its internal structure [22]. Frequent refactoring ensures that the design of a software system stays simple and fit for purpose, without unnecessary complexity or duplication. This helps to prevent errors and facilitates future changes to the system.

**Fig 9:**
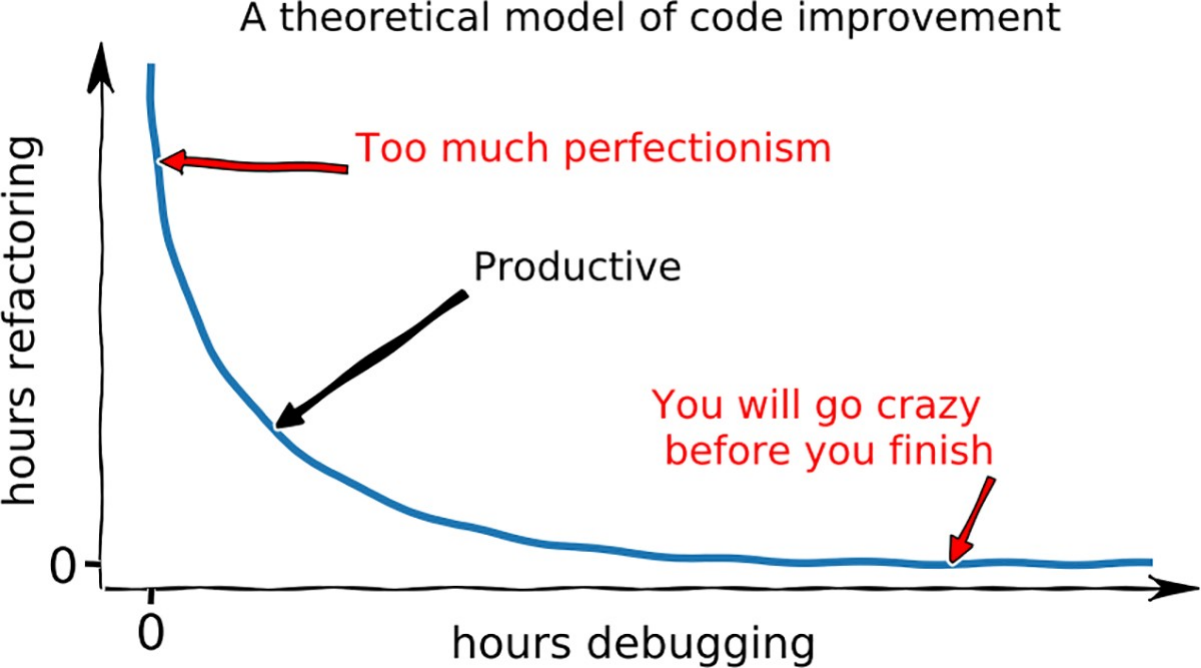
Refactoring can save you from debugging and is also more fun. However, it can also be overdone, so try to find the right balance.

For some scientists, frequent refactoring may be a tall order. After all, the goal of a research project is science and not coding, and if the system ain’t broke, then why fix it? Nevertheless, frequently changing requirements are a common feature of many research projects, and reusing code is more efficient than constantly starting from scratch. Most importantly, a few minutes spent refactoring can save hours of future debugging. So if you find yourself spending too much time on debugging and managing your code, you should consider refactoring more frequently (Fig 9).

A good way to get started with refactoring is to consult lists of “code smells”—general characteristics of code that is unnecessarily complex and could be simplified ([12]; Section 24.2). Examples of such code smells include duplicated code, long functions, long parameter lists, or the need to make synchronized changes at distinct places in your codebase. Many types of code smells can be detected automatically by various tools [28], which can aid you in your programming. You should also be particularly wary of situations where you have developed code with a particular aim and then had to repurpose the code; the assumptions you originally made may no longer be relevant and may need to be refactored out of your software.

To succeed with refactoring, focus on making small changes with little risk of breaking the code or introducing bugs. Some examples of such small and easy refactoring are giving variables better names, making global variables local to a function, eliminating obvious duplicate code, breaking up large functions into smaller ones, grouping related functions into classes, and reorganizing functions and classes into appropriate files. Such changes can be made in a few minutes and will not slow down your coding very much. Furthermore, frequent refactoring in small amounts saves you from having to make larger and riskier changes in the future. In the case that you do need to make larger changes, automated tests can be very helpful, as they can quickly let you know if you have broken something in the code while you refactor. Finally, make sure to version control your code, so that if your refactoring goes badly, you can always go back to your latest working version.

### Rule 8: Write self-documenting code for programmers and a readme file for users

“*It is not enough for code to work*.*”—Robert C*. *Martin*, *Clean Code*

Code documentation is information about a computer program that is intended for human consumption and will not be used by the computer for the program’s execution. It can take the form of external documents, such as user manuals or code vignettes, or code-level information, which are the code itself and inline comments. In the early experimental phases of a research project, there can be a lot of changes to the codebase, and we therefore recommend sticking to code-level documentation in the early phases of your project, rather than creating external documents. This is because it is much easier to keep code-level documentation in-sync with your changes.

The majority of useful code-level information is contained within the code itself. You can maximize this information by using good program structure, clear variable and function names, named constants instead of literal numbers, and by minimizing the complexity of control-flow statements and data structures ([12]; Section 32). Improving your code via refactoring (see Rule 7) is therefore also a form of documentation, and it is better to simplify a complex code, rather than trying to explain it with extensive documentation.

**Fig 10:**
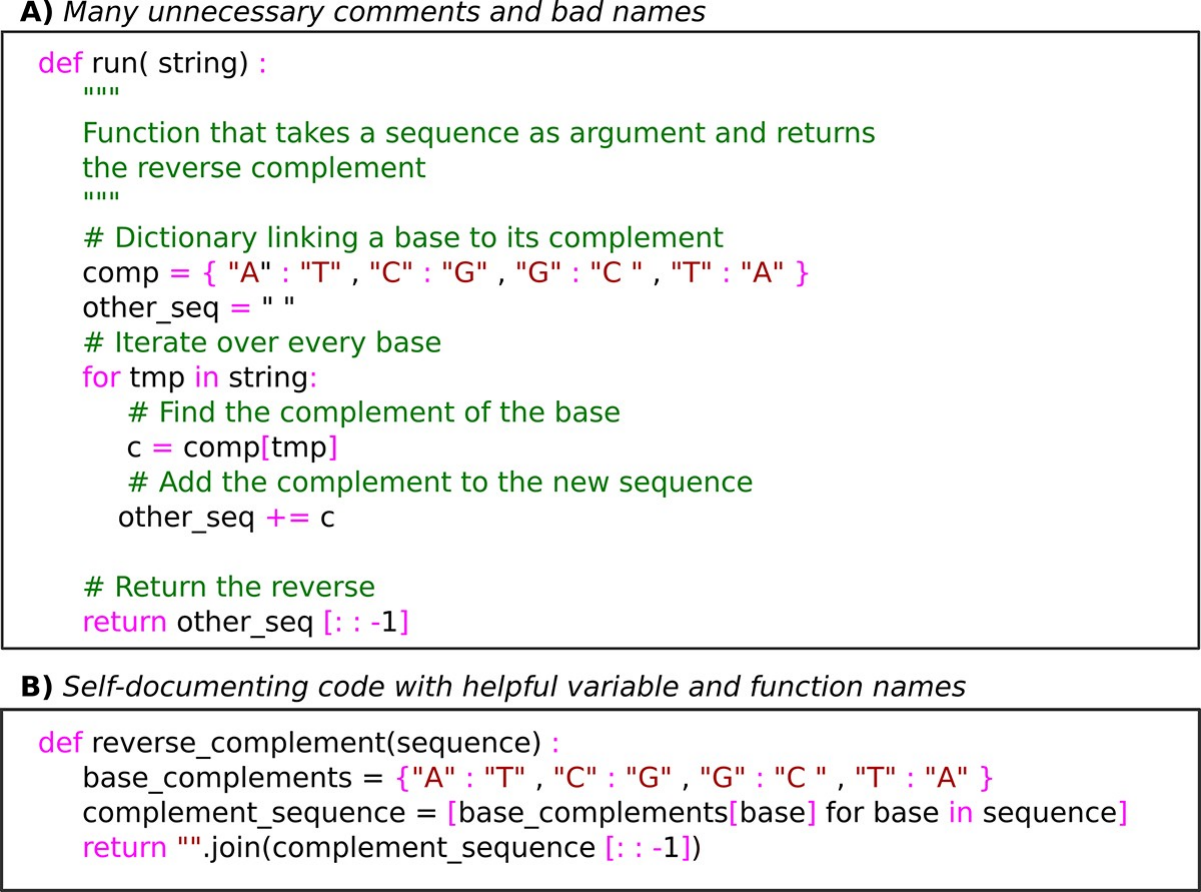
Example Python code written in (A) a bad style with unnecessary comments and in (B) a better self-documenting style.

To increase the readability of your code, try to imitate human sentences. This can be facilitated by using verbs to name your functions, nouns to name your variables, and by giving boolean variables the prefix “is.” An example of bad versus self-documenting code style is given in Fig 10. Finally, do not be afraid to use long names; most modern code editors have auto-complete and search and replace features that make working with long names easy.

The use of in-line comments is a personal choice, but ideally, computer code should explain itself. If you do use in-line comments, try to explain your intentions or summarize your code, rather than repeating the information contained within the code itself ([12]; Section 32). A good trick is to write out your algorithm in pseudocode first, before implementing it in computer code. The pseudocode comments can help you to organize your thoughts and can guide later programmers to the relevant parts of your code if it needs to be updated.

You can use references to external documents, such as published research papers and technical manuals, to further minimize your documentation efforts. This is particularly relevant when implementing mathematical formulas and algorithms. In these situations, try to keep your variable names consistent with the external reference, and note the reference and the location of the relevant algorithm or formula as an inline comment. This minimizes the amount of documentation that you need to write and allows you to use short variable names like “x” and “y” that make your code read like a mathematical equation.

When your computational experiments or analyses have settled into a publishable form, you may want to share some of your code with other researchers by making it publicly available. At this point, changes to your code should be less frequent, and it is worthwhile to create basic external documentation for your “end users,” who will not want to read your code-level documentation. A basic readme file is the quickest way to create external documentation, which will most likely be automatically displayed on your repository pages by code hosting services such as Github. Your readme file should include a statement of purpose, installation instructions, and some simple use-case examples to get your users started.

### Rule 9: Grow your libraries and tools organically from your research

“*A rock pile ceases to be a rock pile the moment a single man contemplates it*, *bearing within him the image of a cathedral*.*”—Antoine de Saint-Exupery*, *The Little Prince*

During the course of a computational research project, you typically write computer code to perform tasks and solve problems. Some of this code could be useful for other research projects, and you may want to store it for future use. In the long run, this can be an incredibly powerful strategy, as it helps you to develop a niche, an area where you can quickly and efficiently carry out research by reusing the fruits of your past projects.

We therefore recommend organizing your code into two categories: project code and library code. Project code should address a specific research question, whereas library code should contain reusable components for ongoing and future projects. This has the benefit of simplifying your research code, as technical tasks such as data handling and preprocessing can be off-loaded to your libraries. You may also want to maintain a set of tools, that is programs with user interfaces for carrying out practical tasks (e.g., a data format conversion). Tools can often be quickly spun out of a library by connecting an appropriate function to a user interface. In most circumstances, we recommend command line interfaces over graphical interfaces, as command line interfaces are technically simpler and allow you to easily chain together your tools via automated scripts.

Library code has higher-quality requirements than project code. This is because research projects are uncertain and typically have limited lifespans, whereas your libraries need to be maintained in the long term. When building a library, you should plan for the possibility of future changes in any dependent software packages. Such changes may force you to perform maintenance work. Prepare for such future changes by being ruthless about eliminating duplicate code, as this simplifies future code updates and makes them less error prone. Also, consider writing automated unit tests for your more critical library components [27].

Due to the higher-quality demands of library code, we recommend being selective about which code you put into a library. Do not try to build your libraries before your research projects, as it is very difficult to predict which software components you will want to reuse. Instead, grow your libraries organically from your research projects, that is, move a software component into a library only after it has proven its worth. For instance, if you find yourself writing the same class in 2 different research projects, you can move the class into a library instead, and use the library in your projects. If you build your libraries in this way, you will only need to maintain the code that you truly need.

### Rule 10: Go explore and be rigorous when you publish

“*The sea is dangerous and its storms terrible*, *but these obstacles have never been sufficient reason to remain ashore*.*”—Ferdinand Magellan*

The rules of this guide are designed to allow you to explore computational ideas in an uncertain environment by programming efficiently. Psychologically, this can allow you to limit your investment in an idea until it proves valuable and to avoid the fallacy of sunk costs [29], which is continuing an endeavor as a result of previously invested resources rather than the endeavor’s merit. The less time and effort that you spend on testing an idea, the easier it will be to change it or give it up in favor of a more fruitful direction.

At the same time, your programming speed must be combined with quality: to avoid misleading errors, false scientific conclusions, and nonreproducible results. Although you can be “quick and dirty” in the early phases of your projects, you should ensure quality and reproducibility when you publish [7]. In particular, you should have a scripted computational workflow that reproduces each publishable result after the exploratory phase of your project is done and your conclusions are drawn. Such scripts can be made using shell programming (Bash), computational notebooks (e.g., Jupyter [30]), or workflow description languages such as NextFlow [31] or Snakemake [32]. Finally, in the very least, your results should be free from programming errors and meet the standards of your field.

## Conclusion

Developing a fast programming style with high-quality code is a lifelong endeavor, and our rules serve as a starting point. To become truly proficient, you should experiment and learn what works for you. We hope that integrating our advice into your programming style will help you to develop as a computational biologist and become better equipped to take on the scientific challenges of your field.
